# The complete mitochondrial genomes of *Pangasius nasutus* and *P. conchophilus* (Siluriformes: Pangasiidae)

**DOI:** 10.1080/23802359.2022.2158694

**Published:** 2023-01-02

**Authors:** Siti Amalia Aisyah Abdul Halim, Yuzine Esa, Han Ming Gan, Amir Asyraf Zainudin, Siti Azizah Mohd Nor

**Affiliations:** a Department of Aquaculture, Faculty of Agriculture, Universiti Putra Malaysia, Serdang, Malaysia; b International Institute of Aquaculture and Aquatic Sciences, Universiti Putra Malaysia, Port Dickson, Malaysia; c Department of Biological Sciences, Sunway University, Petaling Jaya, Malaysia; d Institute of Marine Biotechnology, Universiti Malaysia Terengganu, Kuala Terengganu, Malaysia

**Keywords:** *Pangasius nasutus*, *Pangasius conchophilus*, next-generation sequencing (NGS), mitochondrial genome, phylogenetic

## Abstract

The catfish, *Pangasius nasutus* and *P. conchophilus*, are often misidentified between each other due to their similar morphology. Thus, the current study was conducted to differentiate them based on a molecular approach. The complete mitochondrial genomes of *P. nasutus* and *P. conchophilus* obtained from the Pahang River (Peninsular Malaysia) were sequenced, assembled, and annotated using next-generation sequencing (NGS). A 16,465 bp and 16,470 bp length mitogenome sequence of *P. nasutus* and *P. conchophilus*, respectively, was generated, each containing 13 protein genes, 22 tRNAs, and two rRNAs, typical of most vertebrates. This is the first report of the complete mitochondrial genome sequences of *P. nasutus* and *P. conchophilus*. These data are a valuable genetic resource for future studies of these two commercially important species.

## Introduction

*Pangasius nasutus* (Bleeker 1863) is a commercially important species of catfish in the genus *Pangasius.* In Malaysia, it is locally referred to as 'Patin buah’. It has a disjunctive distribution occurring in Pahang River and its tributaries (Peninsular Malaysia), Batang Rajang (Malaysian Borneo), Batang Hari, Indragiri, Musi (Sumatra) and Kalimantan, Barito, Kahayan, Kapuas (Indonesian Borneo) (Roberts and Vidthayanon 1991; Parenti and Lim 2005; Kottelat 2013; Gustiano 2016). It is a native species in the Pahang River, the largest river in Malaysia. *Pangasius nasutus* is differentiated from all other species in the genus *Pangasius* by having an inferior mouth type, an entirely exposed tooth band of the upper jaw when the jaws are closed, and a strongly projected snout (Roberts and Vidthayanon 1991). *Pangasius conchophilus* Roberts and Vidthayanon 1991, is locally referred to as 'Patin buah kemboja’, and is also considered a commercially important species of catfish in the genus *Pangasius* and constantly misidentified with *P. nasutus.* Unlike the native *P. nasutus*, *P. conchophilus* is an introduced species into Malaysia. Its native origin is from the Mekong, Bangpakong, and Chao Phraya basins (Roberts and Vidthayanon 1991; Kottelat 2013). During the 1990s, *P. conchophilus* fish fries were brought in from Cambodia through Thailand and farmed along the Pahang River by Cambodian immigrants (Baharuddin 2016). *Pangasius conchophilus* is differentiated from all other species in the genus *Pangasius* by having a subterminal mouth, less strongly projecting snout, and large eye diameter (Roberts and Vidthayanon 1991; Baharuddin 2016). Several authors have recognized a very close relationship between *P. nasutus* and *P. conchophilus* and they often misidentified as each other (Roberts and Vidthayanon 1991; Pouyaud et al. 1998; Pouyaud et al. 2000; Baharuddin 2016; Pouyaud et al. 2016; Gustiano et al. 2018). To our knowledge, there is no previous study on the mitogenomes of these two species, which raises questions about their evolutionary relationships. To better understand the taxonomic relationships between the two species, we sequenced, assembled, and annotated the whole mitochondrial genome of *P. nasutus* and *P. conchophilus*, which would serve as a significant genomic resource for future research.

## Materials

**Figure 1.**
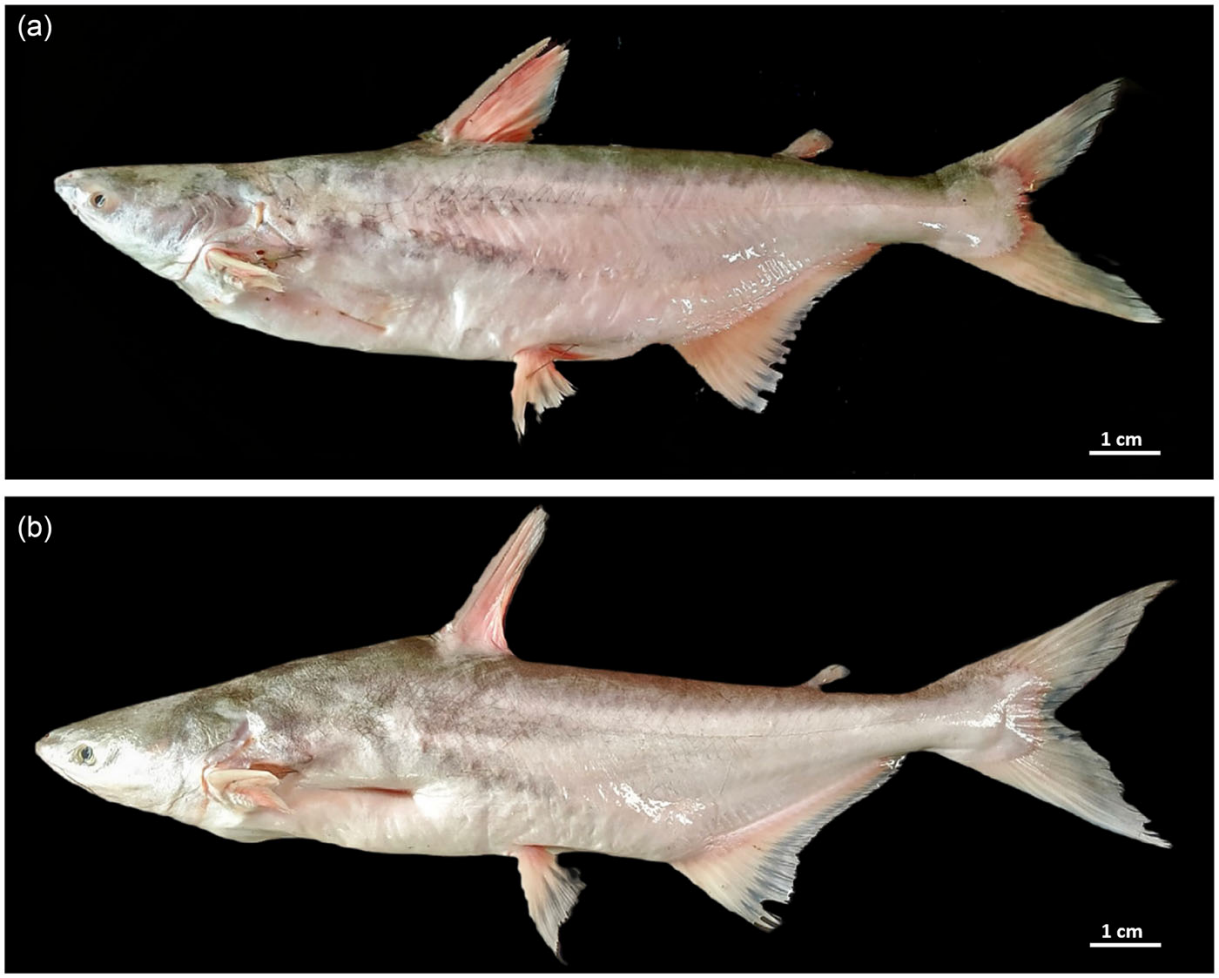
Representative of (a) *Pangasius nasutus* and (b) *Pangasius conchophilus* collected in the study (own photo) (scale = 1 cm).

A single adult specimen of *P. nasutus* (specimen ID: TM15) (Figure 1(a)) was collected from Kuala Krau, Temerloh River (3°42′25.9″N 102°22′25.3″E), while the *P. conchophilus* individual (specimen ID: JR15) (Figure 1(b)) was sampled from Kuala Tembeling, Jerantut River (4°04′13.4″N 102°18′57.1″E). Both Temerloh and Jerantut rivers were part of the Pahang River tributaries. The fish specimens were morphologically identified based on Roberts and Vidthayanon (1991), Kottelat (2013), and Baharuddin (2016). The fish muscle from the caudal peduncle and pectoral fins were excised, preserved in 99% ethanol, and stored at −80 °C until further use for DNA extraction. All specimens are deposited in the Depository Museum, Department of Aquaculture, Faculty of Agriculture, Universiti Putra Malaysia (Dr Zafri Hassan, +60397694932, mzafri@upm.edu.my) under the voucher number JAQ/PN/00001 and JAQ/PC/00001.

## Methods

Approximately, 20–50 mg of tissue sample was used for DNA extraction using WizPrep gDNA Mini Kit (WizBio, Seongnam, South Korea) based on the manufacturer’s instructions. DNA samples were treated with 1 μL of RNAse (10 mg/mL) for 30 min at room temperature, followed by purification using a 1× volume of SPRI bead (Oberacker et al. 2019). Then, 2 μL of the purified DNA was measured using Denovix high sensitivity kit (Denovix, Wilmington, DE). Approximately, 100 ng of DNA was fragmented to 350 bp using a Bioruptor followed by NEB Ultra II library preparation according to the manufacturer’s instructions (NEB, Ipswich, MA). Sequencing was performed on a NovaSEQ6000 (Illumina, San Diego, CA) using a run configuration of 2 × 150 bp to generate approximately 1 Gb of data for each sample. Raw reads were trimmed with fastp v0.21 (https://github.com/OpenGene/fastp.git) (Chen et al. 2018) to remove low-quality bases and Illumina adapter sequences. The trimmed reads were subsequently used for *de novo* assembly in MegaHIT (default setting) (https://github.com/voutcn/megahit.git) (Li et al. 2015). The mitochondrial-derived contigs were identified, circularized, and annotated using MitoZ (https://github.com/linzhi2013/MitoZ.git) (Meng et al. 2019).

To investigate the evolutionary relationships among the *Pangasius* species, the mitogenomes of *P. nasutus* and *P. conchophilus* were aligned with the seven available Pangasiid species mitogenome sequences from GenBank using MUSCLE as implemented in MEGA X (Kumar et al. 2018). The maximum-likelihood (ML) method was employed for phylogenetic reconstruction of the Pangasiid species to determine their relationships. *Ictalurus punctatus* (family Ictaluridae) and *Mystus cavasius* (family Bagridae) were selected as the outgroups. Considering all positions, the ML phylogenetic tree was calculated under the general time reversible (GTR) model using the software RAxML-NG (Kozlov et al. 2019) executed in the graphical interface raxmlGUI 2.0 (Edler et al. 2021). To evaluate the robustness of each node, the bootstrap proportions were computed (500 replicates). The generated tree is displayed in FigTree v1.4.4 (Rambaut 2018).

## Results

**Figure 2.**
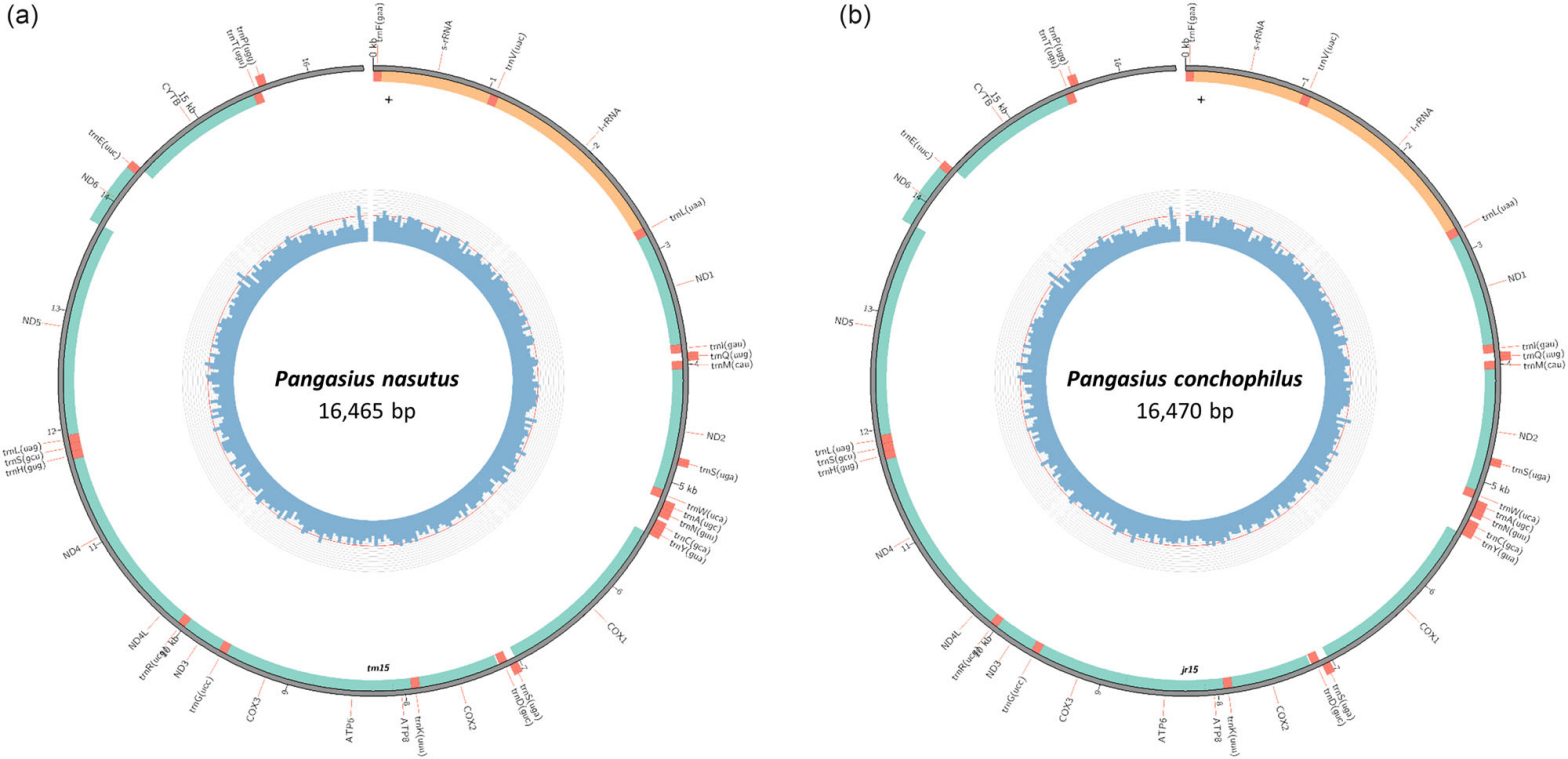
Genome map of (a) *Pangasius nasutus* and (b) *Pangasius conchophilus* mitochondrial genomes.

Both complete mitogenome sequences have been deposited at the National Center for Biotechnology Information GenBank (NCBI) database under the accession numbers OP236030 (*Pangasius nasutus*) (Figure 2(a)) and OP236031 (*P. conchophilus*) (Figure 2(b)). Each mitogenome consists of 37 genes; 13 protein-coding genes (PCGs), 22 transfer RNA (tRNA) genes (duplication of two tRNAs: tRNALeu and tRNASer) on both strands, and two ribosomal RNA (rRNA) genes. The complete mitogenome of *P. nasutus* is a circular molecule with a length of 16,465 bp, and a nucleotide composition of A: 31.3%, T: 26.1%, G: 15.1%, and C: 27.5%. Of the 13 PCGs, only the COX1 gene starts with GTG, while the other 12 genes originate from ATG, in parallel with the other Pangasiid fishes (Jondeung et al. 2007; Wei et al. 2020; Ni et al. 2021). Only Cytb contains an incomplete stop codon (T), while the other genes end with a complete stop codon (TAA or TAG). The complete mitogenome of *P. conchophilus* is 16,470 bp in length, with a nucleotide composition of A: 31.2%, T: 26.1%, G: 15.1%, and C: 27.6%. Similar to *P. nasutus*, of the 13 PCGs, only the CO1 gene starts with GTG, while the remaining 12 genes start with ATG. Furthermore, all PCGs genes contain a complete stop codon (TAA or TAG) except Cytb which contains an incomplete stop codon (T). Based on whole mitogenome alignment, the pair-wise nucleotide similarity between *P. nasutus* and *P. conchophilus* is 0.6%.

**Figure 3.**
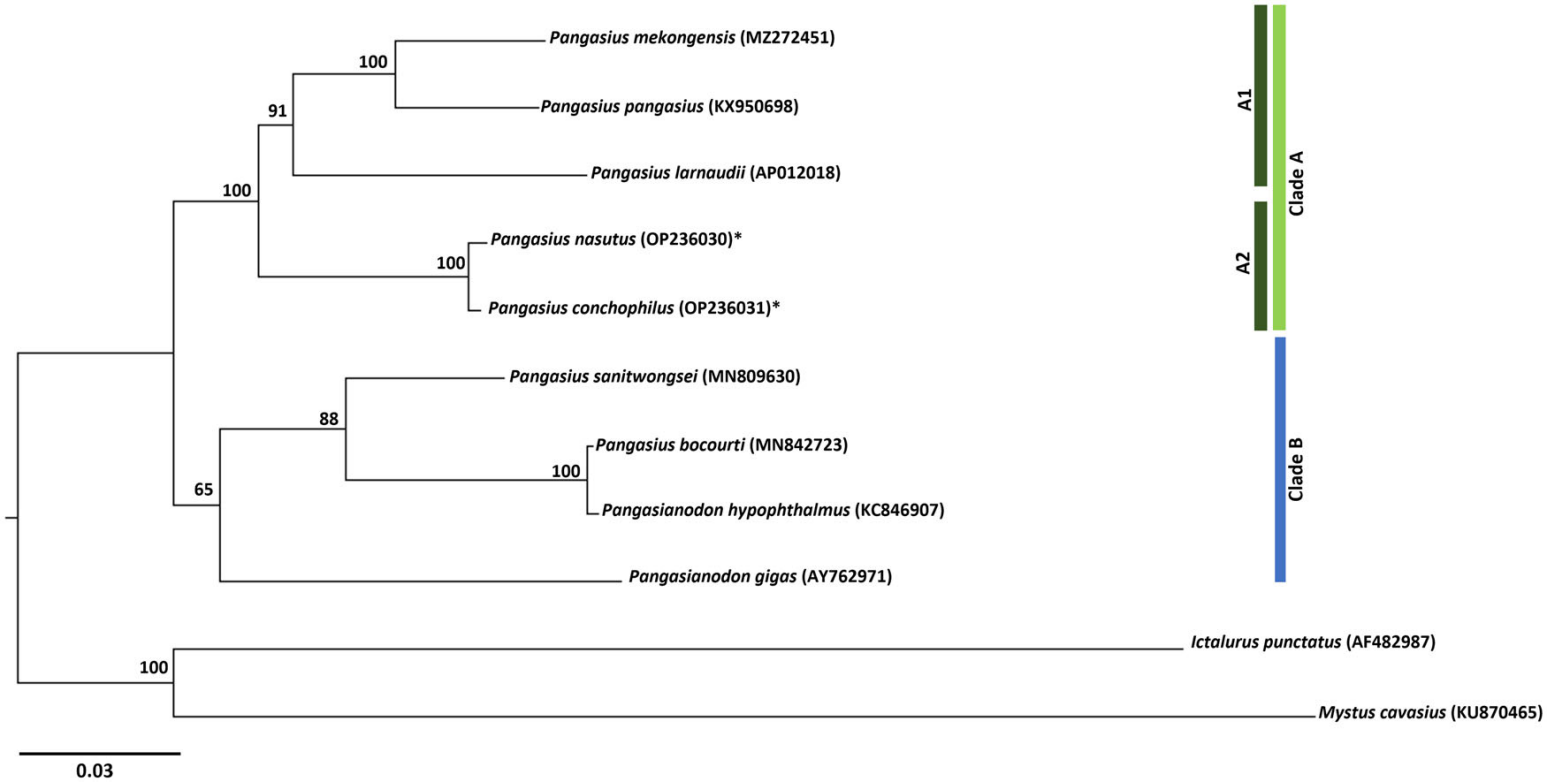
Maximum-likelihood phylogenetic tree of family of Pangasiidae catfishes species based on whole mitogenome sequences. The bootstrap probability values are presented at the nodes. The species study is labeled with asterisks mark *.

The phylogenetic study revealed two main clusters (A and B) (Figure 3). The first cluster bifurcated into two minor clusters (A1 and A2). *Pangasius larnaudii* is closely related to *P. mekongensis* and *P. pangasius*, which is supported by previous research (Ni et al. 2021). In the second minor cluster (A2) *Pangasius nasutus* and *P. conchophilus* form another closely related species and sister to cluster A. The second main cluster (B) shows the close relationship of *P. bocourti* and *Pangasianodon hypophthalmus* forming a sister cluster to *P. sanitwongsei. Pangasianodon gigas* is sister to this cluster in main cluster B, consistent with prior findings (Kim et al. 2018; Chen et al. 2020). Most major and minor clusters are well supported (BP: 100%).

## Discussion and conclusions

We present here the first report of the complete mitochondrial genomes of *Pangasius nasutus* and *P. conchophilus*, having successfully sequenced, assembled, and annotated. The complete structure of the mitogenome published here could be used as a foundation for further research into the population genetics, evolutionary biology, phylogenetic analysis, aquaculture, genomics as well as species identification of Pangasiid catfish and related species.

## Data Availability

The complete mitogenome sequences of *Pangasius nasutus* and *P. conchophilus* have been deposited in the NCBI GenBank database at https://www.ncbi.nlm.nih.gov/ under the accession numbers OP236030 and OP236031, respectively. The associated BioProject, SRA, and Bio-Sample numbers are *Pangasius nasutus*, PRJNA891653, SRR21979525, and SAMN31373448 and for *P. conchophilus*, PRJNA891658, SRR21979584, and SAMN31373450, respectively.
